# Modification of the immunogenicity and antigenicity of rat hepatoma cells. I. Cell-surface stabilization with glutaraldehyde.

**DOI:** 10.1038/bjc.1979.113

**Published:** 1979-06

**Authors:** M. R. Price, R. G. Dennick, R. A. Robins, R. W. Baldwin

## Abstract

gamma-Irradiated rat hepatoma cells are immunogenic in syngeneic WAB/Not rats, so that immunized animals are protected against tumour-cell challenge and circulating tumour-specific antibody is produced. Treatment of the immunizing cells with glutaraldehyde at concentrations of 0.001% or greater for 30 min rendered these cells non-protective in tumour-rejection tests and no longer able to induce significant formation of specific antibody. However, tumour-specific antigens were shown to be expressed upon treated cells; they specifically bound tumour-specific antibody from syngeneic immune sera assessed in indirect membrane-immunofluorescence tests. Also, these cells specifically absorbed antibody from immune or tumour-bearer sera, as demonstrated in the indirect membrane-immunofluorescence test or a complement-dependent 51Cr-release test. Alloantigen expression was not influenced by glutaraldehyde treatment, although glutaraldehyde-treated hepatoma cells failed to induce alloantibody formation in KX/Not rats. Polyacrylamide-gel electrophoresis of treated cells, surface-labelled with 125I, indicated that extensive cross-linking of the surface protein occurred as a result of glutaraldehyde treatment. The present findings establish that although the expression of a tumour-specific antigen is necessary for the induction of immuno-protection against tumour-cell challenge, this alone is not a sufficient condition for eliciting tumour immunity.


*Br. J. Cancer* (1979) **39**, 621

### MODIFICATION OF THE IMMUNOGENICITY AND ANTIGENICITY OF RAT HEPATOMA CELLS. I. CELL-SURFACE STABILIZATION WITH GLUTARALDEHYDE

M. R. PRICE, R. G. DENNICK, R. A. ROBINS AND R. W. BALDWIN

*From the Cancer Research Campaign Laboratories, University of Nottingham, University Park, Nottingham NG7 2RD*

Received 13 December 1978 Accepted 14 February 1979

**Summary.—γ-irradiated rat hepatoma cells are immunogenic in syngeneic WAB/Not rats, so that immunized animals are protected against tumour-cell challenge and circulating tumour-specific antibody is produced. Treatment of the immunizing cells with glutaraldehyde at concentrations of 0·001% or greater for 30 min rendered these cells non-protective in tumour-rejection tests and no longer able to induce significant formation of specific antibody. However, tumour-specific antigens were shown to be expressed upon treated cells; they specifically bound tumour-specific antibody from syngeneic immune sera assessed in indirect membrane-immunofluorescence tests. Also, these cells specifically absorbed antibody from immune or tumour-bearer sera, as demonstrated in the indirect membrane-immunofluorescence test or a complement-dependent $^{51}$Cr-release test. Alloantigen expression was not influenced by glutaraldehyde treatment, although glutaraldehyde-treated hepatoma cells failed to induce alloantibody formation in KX/Not rats. Polyacrylamide-gel electrophoresis of treated cells, surface-labelled with $^{125}$I, indicated that extensive cross-linking of the surface protein occurred as a result of glutaraldehyde treatment.**

**The present findings establish that although the expression of a tumour-specific antigen is necessary for the induction of immuno-protection against tumour-cell challenge, this alone is not a sufficient condition for eliciting tumour immunity.**

THE ATTENUATION of tumour cells by chemical treatment before using them as immunogens has been considered attractive from several viewpoints (Prager & Baechtel, 1973; Sanderson & Frost, 1974; Staab & Anderer, 1977), particularly since previous studies have indicated that immunization with chemically modified antigens, such as flagellin, results in enhanced cell-mediated immunity and depressed antibody responses (*e.g.* Parish & Liew, 1972). Chemical modification may be performed with a high degree of reproducibility, and it is possible to ensure that all cells are rendered inviable. It may be further argued that, by presenting an animal with a chemically attenuated tumour cell, the reactivity against tumour-associated antigens may be enhanced by responses against new determinants introduced by the chemical modification. Indeed, this notion has been supported by experimental evidence ever since Landsteiner (1945) demonstrated that antibodies induced by immunization with conjugated protein had a broad range of specificities. These were determined to be directed against (a) the conjugated group or hapten, (b) determinants of the carrier protein and (c) new determinants composed of the hapten and a portion of the carrier. Other mechanisms have been proposed by which chemical modification may enhance immunogenicity, but without invoking the requisite of the introduction of new groups (Mitchison, 1970).

In the present studies, the effects of glutaraldehyde cross-linking of the cell surface of an aminoazodye-induced rat hepatoma (D23) have been investigated. This tumour is characterized by the expression of a tumour-specific rejection antigen against which it is possible to induce moderate levels of immunoprotection when $\gamma$-irradiated tumour cells are used as immunogen (Baldwin & Barker, 1967*a*; Price *et al.*, 1978). Subcellular preparations or soluble antigen isolated from this hepatoma are generally ineffective in inducing resistance to tumour challenge except when administered within a restricted dose range (Price & Baldwin, 1974; Price *et al.*, 1978). The initial hypothesis considered was to explore whether stabilization of the cell surface with glutaraldehyde as a cross-linking reagent increased or modified the immunogenicity of the tumour, the view being that such treated cells would represent a more persistent immunogen. For this purpose, $\gamma$-irradiated hepatoma cells as an immunogen of defined efficacy were treated with glutaraldehyde at various concentrations and their immunogenicity and expression of surface antigens were evaluated.

### MATERIALS AND METHODS

*Animals, tumours and sera.*—Inbred WAB/Not (Nottingham Cancer Research Campaign Laboratory subline of WAB) and KX/Not (Nottingham Cancer Research Campaign Laboratory subline of KX) rats were maintained by single-line brother–sister mating. Hepatomas D23 and D30, induced by oral administration of 4-dimethylaminoazobenzene, were maintained by serial s.c. passage in WAB/Not rats. Hepatoma D23- and D30-bearer sera were from donors bearing i.p. 9-day implants established by injection of tumour mince (Price & Baldwin, 1977). Hepatoma D23- and D30-immune sera were prepared in syngeneic rats by implantation of $\gamma$-irradiated (15,000R) grafts of tumour. A KX/Not anti-WAB/Not alloantiserum was prepared by s.c. implantation of hepatoma D23 grafts in male KX/Not rats. Serum donors were bled by cardiac puncture under ether anaesthesia, and the serum was collected and stored at −20°C.

*Irradiation of hepatoma D23 cells.*—Single-cell suspensions of hepatoma D23 were prepared by trypsinization of tumour mince and suspended in Hanks' balanced salt solution (HBSS) (Baldwin & Barker, 1967*b*). Tumour cells were attenuated by $^{60}Co$ $\gamma$-irradiation (15,000 R).

*Glutaraldehyde treatment of hepatoma cells.*—Cells suspended in HBSS at $10^7$ cells/ml were diluted with an equal volume of appropriately diluted glutaraldehyde (Sigma Chemical Co., Kingston upon Thames, England) in phosphate-buffered saline, pH 7·3 (PBS). After incubation at room temperature for 30 min, treated cells were sedimented and washed twice with HBSS. In *in vitro* tests, treated cells were resuspended in Eagle's minimal essential medium (MEM) +5% foetal calf serum (FCS) and incubated for at least 30 min before use.

*Indirect membrane-immunofluorescence test.*—This was performed using viable tumour target cells in suspension as previously described (Baldwin & Barker, 1967*b*). A fluorescence index was calculated for test sera by determining the percentage of cells unstained by normal rat serum minus the percentage of cells unstained by the test serum, divided by the former figure, and values of 0·3 or greater were taken to represent a significant membrane immunofluorescence staining reaction (Baldwin & Barker, 1967*b*).

*Radioisotopic antiglobulin test.*—A test was developed to reveal cell-bound, rat alloantibody, using a radioiodinated $F(ab')_2$ fragment of the antibody fraction isolated from a sheep anti-rat IgG antiserum. Briefly, this latter reagent was prepared by ammonium sulphate (33% saturated) precipitation of the Ig fraction from the sheep anti-rat IgG serum followed by pepsin digestion of this fraction (enzyme: substrate, 1:100 in Walpole's acetate buffer, pH 4·5, for 16 h; Stanworth & Turner, 1973). $F(ab')_2$ fragments were then separated by Sephadex G200 column chromatography eluted by upward flow with PBS. The $F(ab')_2$ antibody was then isolated by immunoadsorption to rat IgG convalently linked to CNBr-activated Sepharose 4B (Pharmacia Ltd, Uppsala, Sweden). Bound material was eluted with 50 mM glycine-HCl, pH 2·8, and passed directly over Sephadex G25 to desalt the eluate rapidly. This $F(ab')_2$ antibody preparation was iodinated with $Na^{125}I$ (Radiochemical Centre, Amersham, Bucks) using a modified chloramine-T method (McConahey & Dixon, 1966) to give a specific activity of 0·15 $\mu$Ci/$\mu$g protein

For the isotopic antiglobulin assay, hepatoma D23 cells were seeded into the wells of plastic microtitre plates (Cooke M29 ART) at $10^4$/well, and the plates were incubated at 37°C overnight to allow cell adherence.

After washing the plates by immersion and gentle agitation in 3 separate baths of PBS, cells were pretreated with PBS or 0·01% glutaraldehyde for 30 min at room temperature. The plates were then washed in 3 separate baths of PBS. The contents of each well were then aspirated to leave 90 $\mu$l PBS; 10$\mu$l FCS was added to each well, and incubated at room temperature for 30 min. Aliquots (0·1 ml) of diluted sera were added to each well and, after incubation at 37°C for 30 min, plates were washed in PBS. Aliquots (0·1 ml) of $^{125}$I-F(ab')$_2$ of the antibody fraction of the sheep anti-rat IgG serum (at $10^6$ ct/min/ml) were added to each well and, after incubation at 37°C for 30 min, plates were washed again 4 times with PBS and then air dried. Adherent cells were fixed with Nobecutane aerosol spray (Astra Chemicals Ltd, Watford, England) and individual wells were cut out with a band saw and counted for radioactivity. All PBS solutions used in this assay contained 1·0 mM $Ca^{++}$ and 0·5 mM $Mg^{++}$.

*$^{51}$Chromium-release test.*—This was performed according to a previously described microassay procedure (Price, 1978).

*$^{125}$I-Labelling of cells.*—Hepatoma cell surfaces were radioiodinated according to the method of Phillips & Morrison (1971). Briefly, 200 $\mu$Ci Na$^{125}$I was added to glutaraldehyde-treated or untreated hepatoma cells (1 ml at $10^7$ cells/ml in PBS). To this 50 $\mu$g lactoperoxidase (Sigma Chemical Co., Kingston upon Thames, England) and 25 $\mu$l of 0·03% $H_2O_2$ in PBS was added. The cells were kept at room temperature for 10 min and a further 25 $\mu$l of 0·03% $H_2O_2$ was added. After 10 min, the cells were diluted to 25 ml with PBS and washed ×4 by centrifugation with PBS. The final pellet was suspended in 4 ml of 2% sodium dodecyl sulphate (SDS) containing 2% $\beta$-mercaptoethanol, which was heated to 100°C for 5 min. The resulting solution was dialysed against 0·1% SDS, 0·1% $\beta$-mercaptoethanol in 0·01M sodium phosphate buffer, pH 7·0, for 18 h. Aliquots of the SDS-solubilized cells were separated by analytical polyacrylamide-gel electrophoresis upon 10% acrylamide gels according to the method of Weber & Osborn (1969). Gels were cut into 2 mm slices which were individually counted in an LKB-Wallac Gamma Counter.

### RESULTS

In a preliminary experiment, the dose and timing of i.p. immunization with $\gamma$-irradiated hepatoma D23 cells was investigated. As shown in Table I, treatment of rats with a single injection of $10^6$ immunizing cells from between 28 days before s.c. tumour-cell challenge and 7 days after challenge was ineffective in modifying the incidence of tumours. Immunization with $10^7$ irradiated cells at 7 and 14 days before challenge provided protection against tumour growth and, using the highest immunizing dose of $10^8$ cells, resistance against challenge was evident in all groups of rats receiving immunization up to 28 days before challenge. In no case was it possible significantly to modify the incidence of tumours when treatment was given after tumour-cell challenge (Table I).

In subsequent experiments, when glutaraldehyde $\gamma$-irradiated cells were used as the immunogen, a single dose of $\sim 2{\cdot}5 \times 10^7$ cells was given 7 days before s.c. tumour-cell challenge with between $1 \times 10^3$ and

TABLE I.—*I.p. immunization with $\gamma$-irradiated hepatoma D23 cells—effect of dose and timing*

| Day of immunization before s.c. challenge with $2 \times 10^3$ D23 cells | Tumour takes in 6 rats immunized* with (no. of $\gamma$-IR D23 cells) | | |
|---|---|---|---|
| | $10^6$ | $10^7$ | $10^8$ |
| 28 | 6 | 5 | 3 |
| 21 | 6 | 3 | 0 |
| 14 | 5 | 1 | 1 |
| 7 | 3 | 1 | 0 |
| 3 | 5 | 2 | 1 |
| 0 | 6 | 3 | 2 |
| −3 | 5 | 6 | 6 |
| −7 | 5 | 6 | 5 |

* Tumour takes in untreated, age-matched controls = 22/24.

$5 \times 10^3$ viable D23 cells. These conditions were considered suitable for revealing modification of immunoprotection in rats receiving the chemically modified immunogen.

TABLE II.—*Immunization with glutaraldehyde, γ-irradiated hepatoma D23 cells*

| Immunization* | Tumour incidence in rats challenged with†: | | |
|---|---|---|---|
| | $5 \times 10^3$ cells | $2 \times 10^3$ cells | $1 \times 10^3$ cells |
| None | 5/5 | 6/6 | 10/12 |
| γ-irradiated D23 cells ($2 \cdot 5 \times 10^7$) pretreated with‡: | | | |
| PBS | 1/5 | 1/7 | 2/12 |
| 0·00001% Glutaraldehyde | NT§ | NT | 2/6 |
| 0·0001% Glutaraldehyde | NT | 2/6 | 1/12 |
| 0·001% Glutaraldehyde | NT | 6/6 | 3/12 |
| 0·01% Glutaraldehyde | 5/5 | 6/6 | 8/12 |
| 0·1% Glutaraldehyde | 5/5 | 6/6 | 10/12 |
| 0·5% Glutaraldehyde | 5/5 | NT | NT |
| 2·5% Glutaraldehyde | 5/5 | NT | NT |

* γ-irradiated (15,000 rad) hepatoma D23 cells were injected i.p. 7 days before tumour-cell challenge.

§ Challenged by s.c. injection of tumour cells in 0·2 ml aliquots.

‡ γ-irradiated cells were pretreated for 30 min with glutaraldehyde or, in controls, PBS at room temperature, and they were washed twice by centrifugation with HBSS before injection.

§ NT—Not tested.

Table II shows the results of 3 experiments in which the effect of treating γ-irradiated D23 cells with glutaraldehyde was analysed by determining their capacity to induce immunoprotection against a challenge with D23 cells. It is evident that in rats receiving D23 cells treated with glutaraldehyde at concentrations as low as 0·01% (and in one case 0·001%), the tumour incidence was essentially the same as that in untreated control animals. This contrasts with the result of immunizing rats with γ-irradiated hepatoma cells treated with either PBS or with glutaraldehyde at concentrations lower than 0·001%, when a reduced tumour incidence was apparent.

The humoral response to immunization with glutaraldehyde-treated and untreated γ-irradiated D23 cells, as assessed by the indirect membrane-immunofluorescence test, is shown in Table III. The only group of rats exhibiting a significant membrane-immunofluorescence reaction with sera, giving a fluorescence index $>0 \cdot 3$, was the group immunized by 4 weekly i.p. injections of untreated, γ-irradiated D23 cells (Group 2, Table III). The capacity of these cells to induce specific antibody production was abolished by pretreatment with glutaraldehyde at concentrations of 0·001 and 0·01% (Groups 3 and 4, respectively; Table III).

Although these results suggest that the activity of the tumour-specific antigen associated with D23 is lost or significantly diminished when assayed by the induction of immune responses, the results in Table IV indicate that glutaraldehyde-treated γ-irradiated D23 cells still retain a serologically identifiable tumour-specific antigen when assessed in membrane-immunofluorescence tests. In these experiments, D23 immune or bearer serum (containing antibody specifically reactive with D23 cells (Baldwin & Barker, 1967*a*; Price & Baldwin, 1977) reacted positively with γ-irradiated D23 cells, giving fluorescence

TABLE III.—*Humoral response to immunization with glutaraldehyde-treated γ-irradiated D23 cells*

| Group | Immunization procedure* | % Glutaraldehyde† | Fluorescence index) (mean ± s.d.) |
|---|---|---|---|
| 1 | 1 ml HBSS × 4 | — | 0·04 ± 0·03 |
| 2 | $10^7$ γ-irradiated D23 cells × 4 | — | 0·48 ± 0·11 |
| 3 | $10^7$ γ-irradiated D23 cells × 4 | 0·001 | 0·11 ± 0·16 |
| 4 | $10^7$ γ-irradiated D23 cells × 4 | 0·01 | 0·08 ± 0·11 |

* Immunizing γ-irradiated (15,000 rad) D23 cells were injected i.p. at weekly intervals. Rats were bled 7 days after the final injection.

† Cells were pretreated for 30 min with glutaraldehyde or, in controls, with PBS at room temperature and washed twice by centrifugation with HBSS before injection.

TABLE IV.—*Membrane-immunofluorescence reactions with glutaraldehyde-treated γ-irradiated D23 cells*

| Serum | Fluorescence indices (mean ± s.d.) with γ-irradiated D23 cells pretreated* with glutaraldehyde at a concentration of: 0% | 0·001% | 0·01% |
|---|---|---|---|
| Normal WAB/Not serum | — | 0·02 ± 0·03 | 0·09 ± 0·06 |
| D23 IR graft-immune | 0·49 ± 0·06 | 0·54 | 0·54 ± 0·05 |
| D30 IR graft-immune | 0·03 ± 0·00 | 0·14 | 0·16 ± 0·08 |
| D23-bearer serum | 0·57 ± 0·06 | 0·55 ± 0·05 | 0·61 ± 0·05 |
| D30-bearer serum | 0·06 ± 0·05 | 0·05 ± 0·02 | 0·09 ± 0·06 |
| KX/Not anti-WAB/Not alloantiserum | 1·00 ± 0·00 | 1·00 ± 0·00 | 1·00 ± 0·00 |

* For 30 min at room temperature.

TABLE V.—*Absorption of anti-D23 antibody by glutaraldehyde-treated cells: membrane immunofluorescence tests*

| Absorption conditions*: Expt. | Absorbing tumour | No. cells/ml serum ($10^7$) | % Glutaraldehyde† | Fluorescence index (mean ± s.d.) |
|---|---|---|---|---|
| 1 | — | — | — | 0·55 ± 0·06 |
| | D23 | 2 | — | 0·14 ± 0·07 |
| | | 2 | 0·01 | 0·14 ± 0·07 |
| | | 5 | — | 0·05 ± 0·05 |
| | | 5 | 0·01 | 0·00 ± 0·05 |
| | D30 | 2 | — | 0·42 |
| | | 2 | 0·01 | 0·49 |
| | | 5 | — | 0·46 |
| | | 5 | 0·01 | 0·46 |
| 2 | — | — | — | 0·64 ± 0·04 |
| | D23 | 2 | — | 0·14 ± 0·09 |
| | | 2 | 0·5 | 0·29 ± 0·06 |
| | | 5 | — | 0·07 ± 0·06 |
| | | 5 | 0·05 | 0·13 ± 0·08 |
| | D30 | 2 | — | 0·59 ± 0·04 |
| | | 2 | 0·5 | 0·57 ± 0·04 |
| | | 5 | — | 0·56 ± 0·09 |
| | | 5 | 0·5 | 0·56 ± 0·07 |

* At 4°C for 2 h.
† Pretreatment for 30 min at room temperature.

TABLE VI.—*Absorption of anti-D23 antibody by glutaraldehyde-treated cells: complement-dependent cytotoxicity test*

| Absorption conditions*: Absorbing tumour | No. cells/ml serum ($10^7$) | % Glutaraldehyde† | % Cytotoxicity after 2 h | 4 h |
|---|---|---|---|---|
| — | — | — | 40·2 (3·0)‡ | 46·2 (1·9) |
| D23 | 2 | — | 4·7 (0·7) | 6·3 (−0·2) |
| | 2 | 0·5 | 6·3 (−0·6) | 8·3 (4·0) |
| | 5 | — | 4·7 (−1·5) | 7·9 (−0·4) |
| | 5 | 0·5 | 6·3 (−0·6) | 8·1 (1·0) |
| D30 | 2 | — | 38·1 (0·9) | 42·3 (1·8) |
| | 2 | 0·5 | 35·4 (1·2) | 38·9 (1·1) |
| | 5 | — | 37·1 (0·8) | 40·1 (2·5) |
| | 5 | 0·5 | 32·1 (0·4) | 38·9 (−1·3) |

* At 4°C for 2 h.
† Pretreatment for 30 min at room temperature.
‡ In parentheses, the % cytotoxicity in samples to which heat-inactivated complement (56°C for 60 min) was added.

indices of >0·3, and these reactions were demonstrable using glutaraldehyde-treated as well as untreated tumour target cells. Conversely, sera from rats sensitized to hepatoma D30 (*i.e.* from donors immunized against, or bearing this tumour) failed to react with treated or untreated D23 target cells (Table IV).

That glutaraldehyde failed to modify the reactivity of D23-specific antigen in *in vitro* tests is further confirmed in Tables V and VI. These tests showed that absorption of sera containing specific antibody to D23 with glutaraldehyde-treated and untreated D23 cells removed their reactivity for D23 target cells, as assessed by the indirect membrane-immunofluorescence test (Table V) and by complement-dependent cytotoxicity assays using a $^{51}$Cr-release test (Table VI). Exposure of cells to concentrations of glutaraldehyde as high as 0·5% did not modify their capacity to remove tumour-specific antibodies. However, when these sera were absorbed with treated or untreated D30 cells, their reactivities for D23 cells by immunofluorescence (Table V) or complement-dependent cytotoxicity (Table VI) were equal, and in all cases comparable to those of unabsorbed sera.

It is clear from the data in Table IV that KX/Not anti-WAB/Not alloantigens associated with D23 were largely unaffected by treatment with glutaraldehyde, since this alloantiserum reacted strongly with treated and untreated target cells, giving in all instances a fluorescence index of 1·0. This conclusion was substantiated by a more quantitative radioisotopic antiglobulin assay using an $^{125}$I-labelled F(ab')$_2$ fragment of the antibody from a sheep anti-rat IgG serum to reveal cell-surface-bound rat immunoglobulin on D23 cells. As shown in Table VII, the uptake of the antiglobulin reagent was essentially equal with both untreated D23 cells and with D23 cells treated with 0·1% glutaraldehyde, which had been exposed to normal or alloantiserum at dilutions of 1/100, 1/500 and 1/1000. At each of these dilutions the uptake of radiolabel in cells exposed to alloantiserum was considerably greater than in cells exposed to normal serum at the same dilution, or medium alone (Table VII). Although KX/Not anti-WAB/Not alloantigens were still expressed on D23 cells treated with glutaraldehyde, these cells did not induce alloantibody formation in KX/Not rats as assayed by either the indirect membrane-immunofluorescence test of the radioisotopic antiglobulin assay. As shown in Table VIII, serum from KX/Not rats immunized by 4 injections of $10^7$ $\gamma$-irradiated D23 cells (Group 2) showed positive membrane-immunofluorescence staining of D23 cells (fluorescence index, 1·0±0·00), whereas sera from rats immunized with glutaraldehyde-treated D23 cells (Group 3) displayed equal reactivity to the serum from medium-treated donors

TABLE VII.—*Radioisotopic antiglobulin test for the detection of KX/Not alloanti-WAB/Not antibodies bound to glutaraldehyde-treated D23 cells*

| Serum | Dilution | Uptake of $^{125}$I-F(ab')$_2$ antibody of sheep anti-rat IgG serum to: Untreated D23 cells | | D23 cells pretreated* with 0·01% glutaraldehyde | |
|---|---|---|---|---|---|
| Medium | | 19±10† | (49±7) | 0±6 | (57±6) |
| Normal KX/Not serum | 1/100 | 57±59 | (54±10) | 7±11 | (96±13) |
| | 1/500 | 17±8 | (47±6) | 3±1 | (60±6) |
| | 1/1000 | 10±1 | (56±3) | 8±11 | (53±4) |
| KX/Not anti-WAB/Not alloantiserum | 1/100 | 1141±13 | (107±18) | 1175±27 | (134±4) |
| | 1/500 | 575±129 | (60±1) | 443±107 | (80±22) |
| | 1/1000 | 267±10 | (62±3) | 225±15 | (55±13) |

* For 30 min at room temperature.
† Mean ct/min±s.d. after subtraction of values in wells without added cells (the values in parentheses).

TABLE VIII.—*Induction of KX/Not anti-D23 alloantibody by immunization with glutaraldehyde-treated γ-irradiated D23 cells*

| | | Radioisotopic antiglobulin test | | Membrane-immunofluorescence test |
|---|---|---|---|---|
| Group | Immunization procedure* | Serum dilution | Uptake of $^{125}$I-F(ab′)$_2$ antibody of sheep anti-rat IgG serum† | Fluorescence index (mean ± s.d.) |
| 1 | 1 ml HBSS × 4 | 1/20 | 606 ± 177 | −0·02 ± 0·02 |
| | | 1/200 | 110 ± 126 | |
| | | 1/1000 | 132 ± 44 | |
| 2 | $10^7$ γ-irradiated D23 cells/PBS × 4 | 1/20 | 4612 ± 1434 | 1·00 ± 0·00 |
| | | 1/200 | 1452 ± 655 | |
| | | 1/1000 | 423 ± 105 | |
| 3 | $10^7$ γ-irradiated D 23 cells/0·01% glutaraldehyde × 4 | 1/20 | 465 ± 194 | 0·03 ± 0·05 |
| | | 1/200 | 227 ± 73 | |
| | | 1/1000 | 162 ± 65 | |

* Immunizing, γ-irradiated D23 cells were injected i.p. into groups of 3 rats, × 4 at weekly intervals. Rats bled 7 days after the final injection. γ-irradiated cells were pretreated for 30 min with glutaraldehyde, or in controls, PBS, at room temperature and then washed twice by centrifugation with HBSS before injection.
† Mean ct/min ± s.d. after subtraction of values obtained for wells without added cells.

FIG. — SDS - polyacrylamide - gel electrophoresis of $^{125}$I-labelled surface proteins of untreated hepatoma D23 cells (■——■) and hepatoma D23 cells treated with 0·01% (▲——▲) and 0·5% (●——●) glutaraldehyde. Marker proteins used for mol. wt calibration of the gels were bovine serum albumin, 68,000; ovalbumin, 43,000; pepsin, 35,000; cytochrome c, 11,700. The position of the bromophenol blue (BPB) tracker dye is indicated.

(Group 1). These findings were substantiated by the radioisotopic antiglobulin test (Table VIII) and again the only serum to show greater binding to target cells at dilutions from 1/20 to 1/1000 than control serum at the same dilutions (Group 1) was that obtained from donors immunized with untreated γ-irradiated D23 cells (Group 2).

Finally, the effect of glutaraldehyde treatment of γ-irradiated D23 cells was analysed by gel electrophoresis after lactoperoxidase-catalysed radioiodination of treated cell surfaces. D23 cells, treated either with PBS alone or 0·01% or 0·5% glutaraldehyde, were solubilized by boiling in 2% SDS for 5 min and samples were applied to 10% polyacrylamide gels. The Figure shows the separation achieved, and 2 major features are evident. Firstly, with increased glutaraldehyde concentration, more of the labelled material failed to enter the gel, remaining on the top. Secondly, a similar distribution of labelled proteins was found in each of the gels, indicating that cross-linking of surface proteins was incomplete, even at the higher concentration of glutaraldehyde used (0·5%).

### DISCUSSION

The present findings demonstrate that glutaraldehyde fixation of tumour-cell surfaces renders these cells no longer effective in eliciting immunity to tumourcell challenge, or able to induce tumour-specific or alloantibody formation. However, glutaraldehyde treatment did not apparently modify the ability of these cells to bind either tumour-specific antibody from syngeneic immune sera or KX/Not anti-hepatoma D23 alloantibody, indicating that these antigens associated with this hepatoma were not inactivated. These observations may have several interpretations, and are highly relevant to defining the parameters requisite for the full expression of immunogenicity. Firstly, it may be argued that the serologically defined tumour-specific antigen associated with hepatoma D23 may be different from the determinant eliciting immunity (the latter being labile to glutaraldehyde). This would seem unlikely, since close correlation has been found between the expression and specificity of rejection antigens and antigens identified by humoral and cell-mediated immune assays (reviewed in Baldwin, 1973; Baldwin & Price, 1975) although formal proof of this point is not available. Alternatively, although the presence of a tumour-specific antigen at the cell surface may be necessary for the induction of immunity, that in itself may not be sufficient. Similar proposals have been reported from studies on murine alloantigens, showing that whilst mild treatment with glutaraldehyde did not markedly alter the antigenicity of P815 murine mastocytoma cells, the immunogenicity of these cells was lost (Bubbers & Henney, 1975). Thus, treated cells failed to induce blastogenesis of co-cultivated allogeneic lymphocytes, and cytolytically active cells did not develop in these cultures.

In another investigation, Forman (1977) was able to define the conditions for glutaraldehyde treatment under which treated cells stimulated cell-mediated lympholysis but not mixed-lymphocyte reactivity, demonstrating that the 2 phenomena can be dissociated.

If, as seems likely, the induction of cytotoxic T cells is important for the establishment of tumour immunity (Baldwin, 1978), it may be relevant to consider the possibility that the tumour-specific antigens on glutaraldehyde-treated hepatoma cells are no longer immunogenic because they are unable to generate these effector cells. Indeed, Dennert (1974) has found that glutaraldehyde-treated P815 mastocytoma cells, whilst unable to generate cytotoxic T cells *in vivo*, do give rise to T helper cells. Thus, spleen cells from allogenic C57BL/6 mice immunized with fixed P815 cells displayed no cytotoxicity towards homologous target cells, but formed antitrinitrophenol antibody *in vitro* when incubated with trinitrophenylated P815 cells.

One important factor influencing the immunogenicity of living allogeneic cells has been shown to be the metabolic state of the cells. Indeed, for the *in vitro* induction of cell-mediated cytolysis an active cell metabolism was required, one interpretation being that protein turnover at the cell surface was necessary for the full expression of immunogenicity (Wagner, 1973). To what extent the irradiated hepatoma cells used in the present studies retain an active cell metabolism, albeit for a limited period, is the subject of a later report (Dennick *et al.*, 1979). The effects of glutaraldehyde on cell metabolism are profound, and in one study it was found that treatment with 0·15% glutaraldehyde for only 10 s destroyed cells' ability to incorporate amino acids or nucleotides into macromolecules (Bubbers & Henney, 1975). Certainly the conditions used in the present investigation, involving treatment for 30 min, would be sufficient to modify the efficiency of the cell's synthetic apparatus. Indeed, the conditions of treatment were sufficient to markedly cross-link much of the surface protein, as demonstrated by the polyacrylamide-gel electrophoresis of radioiodinated, glutaraldehyde-treated hepatoma cells (Fig. 1). In these tests, with increasing glutaraldehyde concentrations, more of the labelled material was covalently linked into insoluble high-mol.-wt aggregates which failed to enter the polyacrylamide gel. However, whether there is any parallel between the extent of cross-linking and the reduction in immunogenicity remains to be seen. Also, with other tumour models it has been possible to use various chemical treatments, including the use of cross-linking reagents, for the attenuation of viable tumour cells with retention of demonstrable levels of immunogenicity (Prager & Baechtel, 1973).

Finally, while the glutaraldehyde-fixed cell (expressing the tumour-specific antigen on an essentially inert carrier) may represent a more persistent immunogen, such material may not however be accessible to effective macrophage processing. The processing of different materials such as soluble and particulate antigens is known to differ (Unanue, 1972) and if macrophage degradation of multideterminant cellular antigen is necessary for the induction of immunity (Brunda & Raffel, 1977) clearly the fixed cell as an immunogen would be far less amenable to these processes than the unfixed cell.

This study was supported by the Cancer Research Campaign and by a Government Equipment Grant obtained through the Royal Society.

The authors acknowledge with thanks the skilful technical assistance of Mrs C. Arlen, Mrs C. Wright and Mr O. F. H. Roberts. Mrs M. E. Addison and the staff of the Cancer Research Animal Unit are thanked for the provision and maintenance of animals.

### REFERENCES

BALDWIN, R. W. (1973) Immunological aspects of chemical carcinogenesis. *Adv. Cancer Res.*, **18**, 1.

BALDWIN, R. W. (1978) Immune responses to tumour specific and embryonic antigens on chemically-induced tumours. *Prog. Immunol.*, **3**, 538.

BALDWIN, R. W. & BARKER, C. R. (1967*a*) Demonstration of tumour-specific humoral antibody against aminoazo dye-induced rat hepatoma. *Br. J. Cancer*, **21**, 793.

BALDWIN, R. W. & BARKER, C. R. (1967*b*) Tumour-specific antigenicity of aminoazo dye-induced rat hepatomas. *Int. J. Cancer*, **2**, 355.

BALDWIN, R. W. & PRICE, M. R. (1975) Neoantigen expression in chemical carcinogenesis. In *Cancer: A Comprehensive Treatise*. Ed. F. F. Becker. Vol. 1. New York: Plenum Press. p. 353.

BRUNDA, M. J. & RAFFEL, S. (1977) Macrophage processing of antigen for induction of tumour immunity. *Cancer Res.*, **37**, 1838.

BUBBERS, J. E. & HENNEY, C. (1975) Studies on the synthetic capacity and antigenic expression of glutaraldehyde-fixed target cells. *J. Immunol.*, **114**, 1126.

DENNERT, G. (1974) Evidence for non-identity of T killer and T helper cells sensitivity to alloantigenic cell antigens. *Nature*, **249**, 358.

DENNICK, R. G., PRICE, M. R. & BALDWIN, R. W. (1979) Modification of the immunogenicity and antigenicity of rat hepatoma cells. II. Effects of mild heat treatment. *Br. J. Cancer*, **39**, 630.

FORMAN, J. (1977) T cell-mediated cytotoxicity against trinitrophenyl-modified cells: Effect of glutaraldehyde treatment on the immunogenicity and antigenicity of trinitrophenyl-modified cells. *J. Immunol.*, **118**, 1755.

LANDSTEINER, K. (1945) *Specificity of Serological Reactions*. Cambridge, Massachusetts: Harvard University Press.

MCCONAHEY, P. J. & DIXON, F. J. (1966) A method of trace iodination of proteins for immunological studies. *Int. Arch. Allergy*, **29**, 185.

MITCHISON, N. A. (1970) Immunological approach to cancer. *Transplant. Proc.*, **2**, 92.

PARISH, C. R. & LIEW, F. Y. (1972) Immune response to chemically modified flagellin. III. Enhanced cell-mediated immunity during high and low zone antibody tolerance to flagellin. *J. Exp. Med.*, **135**, 298.

PHILLIPS, D. R. & MORRISON, M. (1971) Exposed protein on the intact human erythrocyte. *Biochemistry*, **10**, 1766.

PRAGER, M. D. & BAECHTEL, F. S. (1973) Methods for modification of cancer cells to enhance their immunogenicity. In *Methods in Cancer Research*. Ed. H. Busch, Vol. 9. New York: Academic Press. p. 339.

PRICE, M. R. (1978) A microassay for the detection of tumour specific complement dependent serum cytotoxicity against a chemically induced rat hepatoma. *Transplantation*, **25**, 224.

PRICE, M. R. & BALDWIN, R. W. (1974) Immunogenic properties of rat hepatoma subcellular fractions. *Br. J. Cancer*, **30**, 394.

PRICE, M. R. & BALDWIN, R. W. (1977) Tumour-specific complement-dependent serum cytotoxicity against a chemically-induced rat hepatoma. *Int. J. Cancer*, **20**, 284.

PRICE, M. R., PRESTON, V. E., ROBINS, R. A., ZÖLLER, M. & BALDWIN, R. W. (1978) Induction of immunity to chemically-induced rat tumours by cellular or soluble antigens. *Cancer Immunol. Immunother.*, **3**, 247.

SANDERSON, C. J. & FROST, P. (1974) The induction of tumour immunity in mice using glutaraldehyde-treated tumour cells. *Nature*, **248**, 690.

STAAB, H.-J. & ANDERER, F. A. (1977) Immunogenicity of tumour cells modified with various chemicals. *Br. J. Cancer*, **35**, 395.

STANWORTH, D. R. & TURNER, M. W. (1973) Immunochemical analysis of immunoglobulins and their subunits. In *Handbook of Experimental Immunology*. Ed. D. M. Weir. Oxford: Blackwell. 2nd Edn. p. 10.

UNANUE, E. R. (1972) The regulatory role of macrophages in antigenic stimulation. *Adv. Immunol.*, **15**, 95.

WAGNER, H. (1973) Cell-mediated immune response *in vitro*. IV. Metabolic studies on cellular immunogenicity. *Eur. J. Immunol.*, **3**, 84.

WEBER, K. & OSBORN, M. (1969) The reliability of molecular weight determination by dodecyl sulphate–polyacrylamide gel electrophoresis. *J. Biol. Chem.*, **244**, 4406.


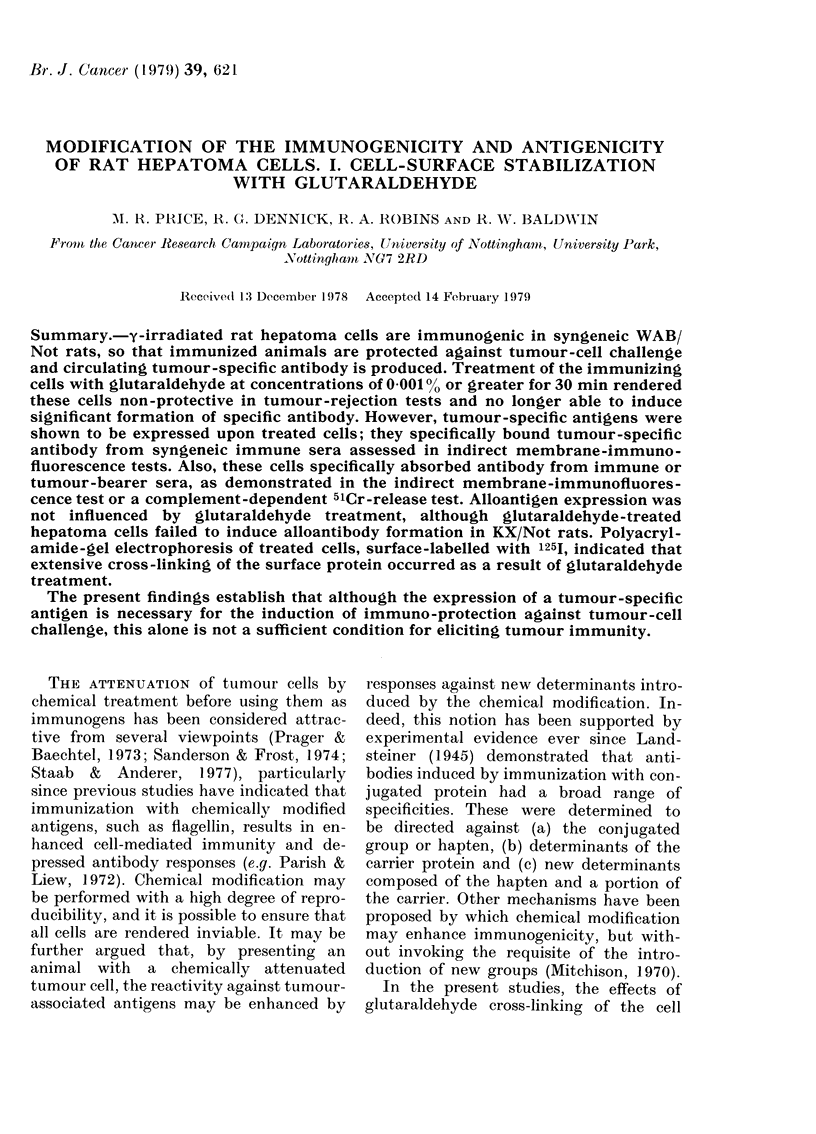

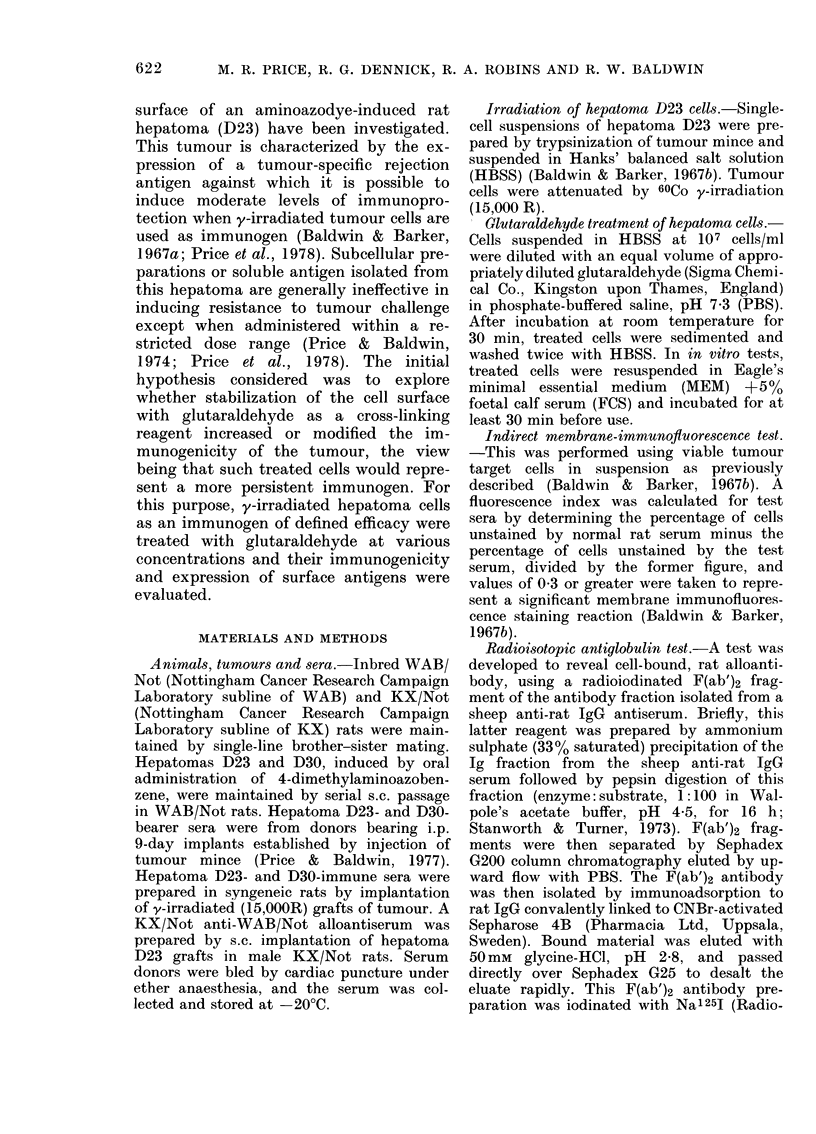

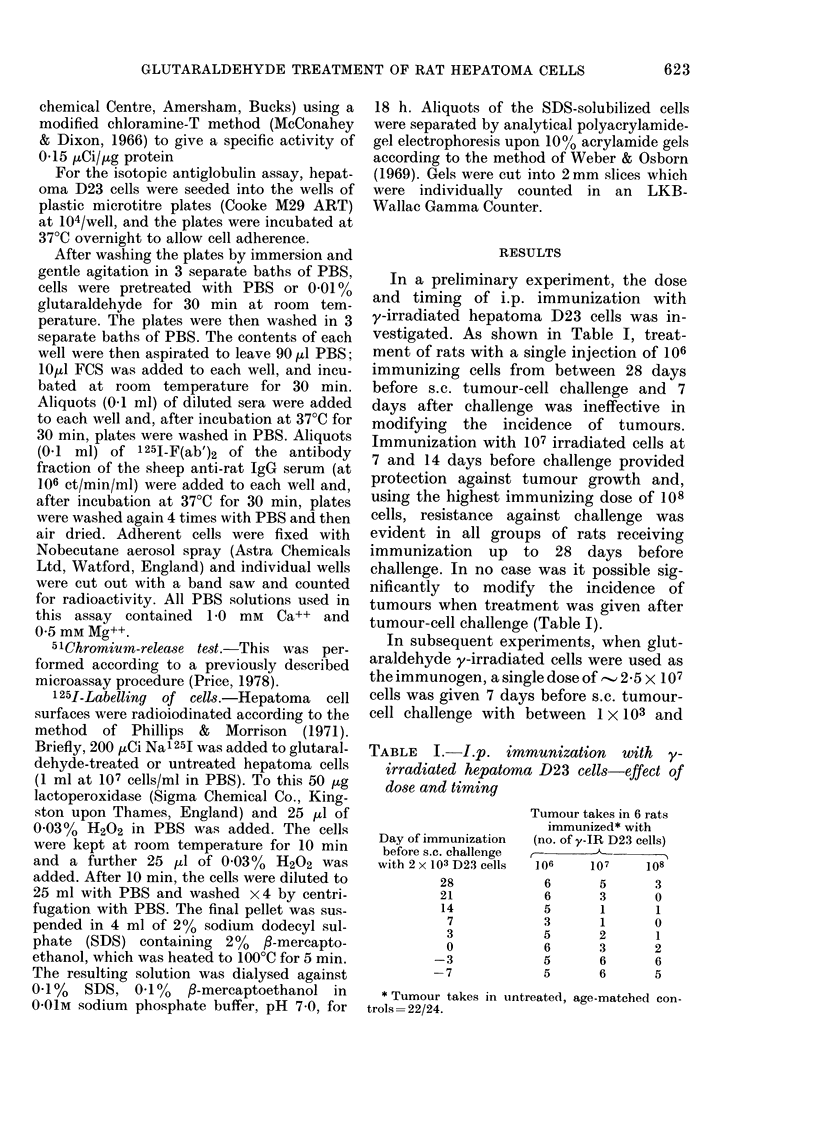

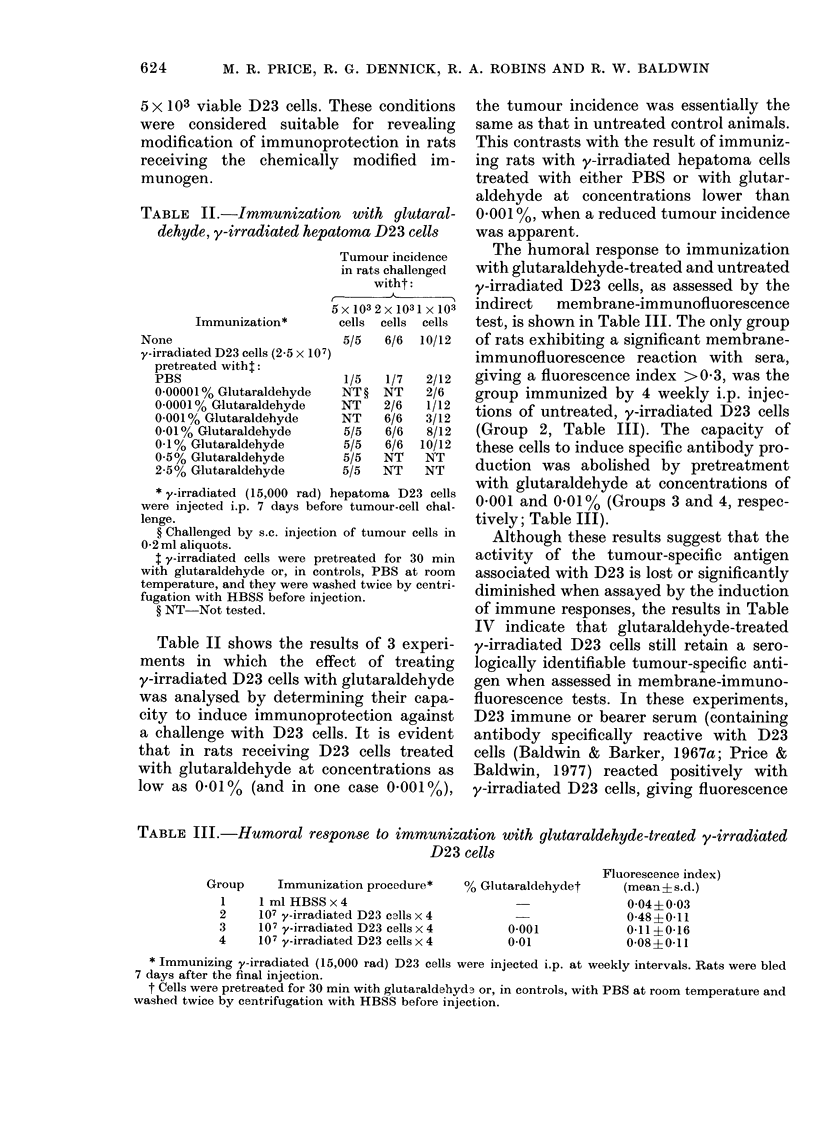

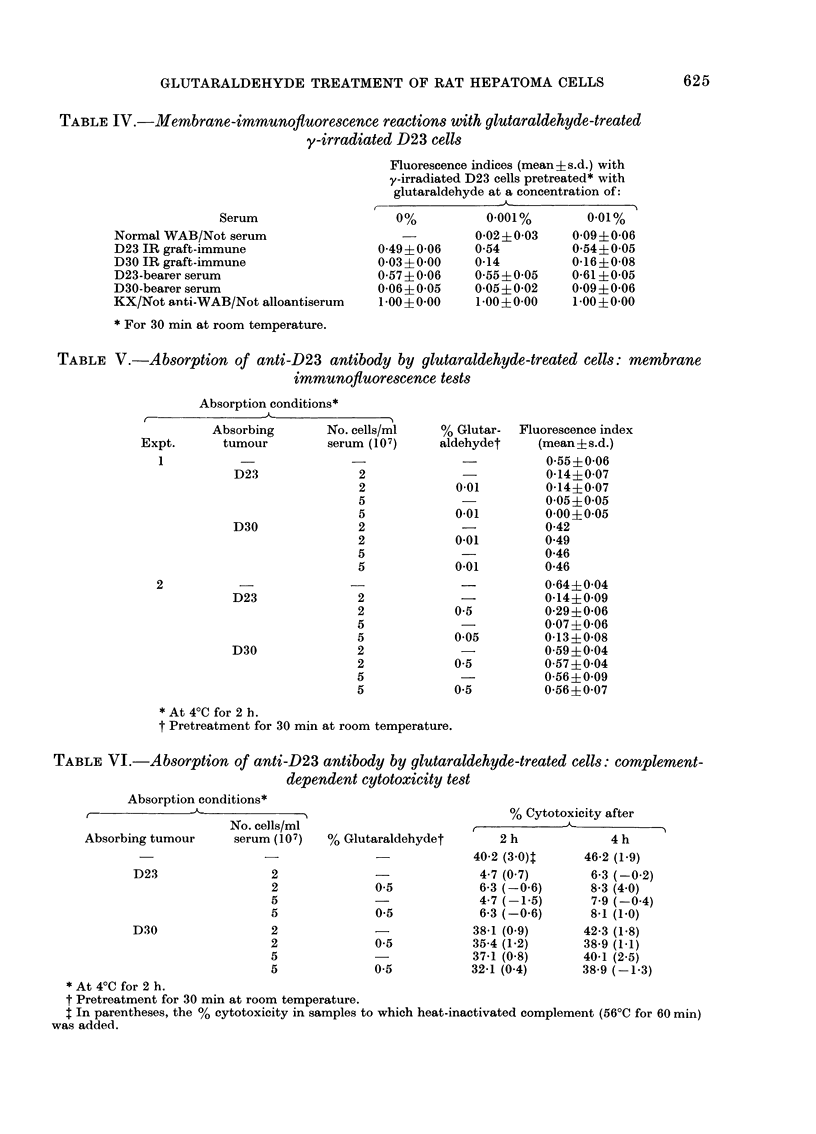

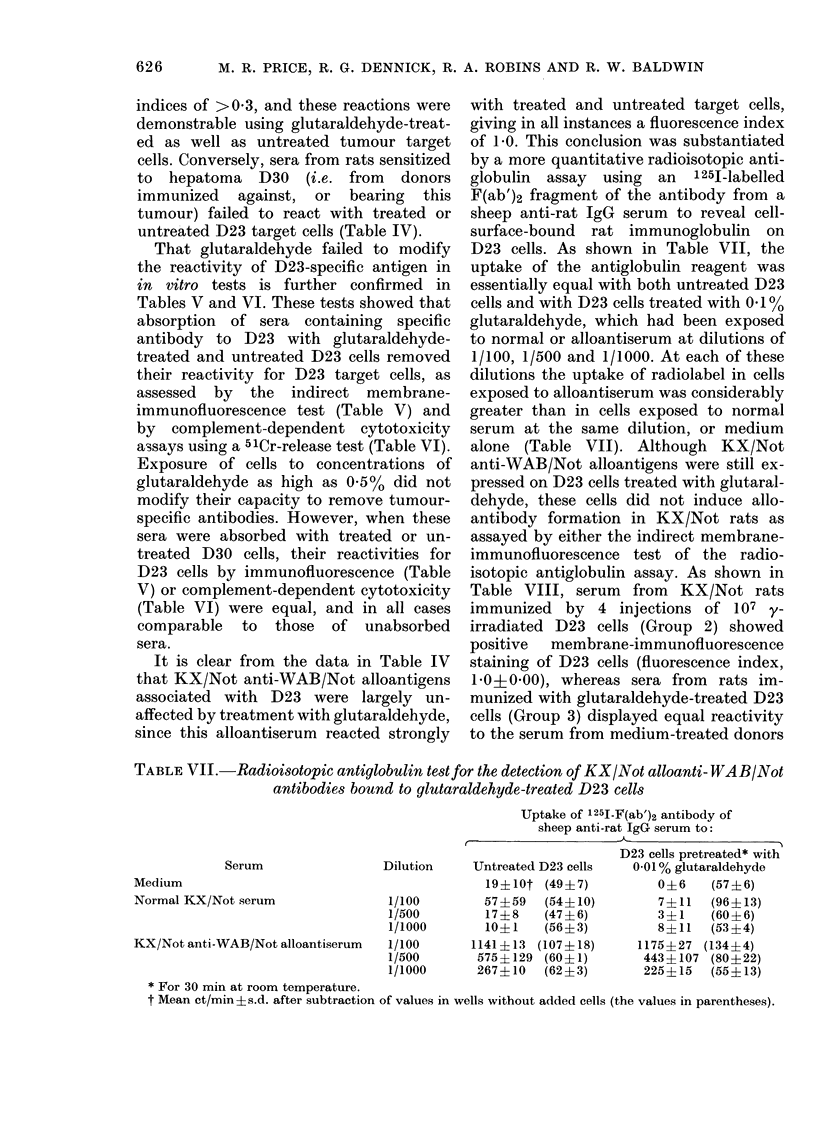

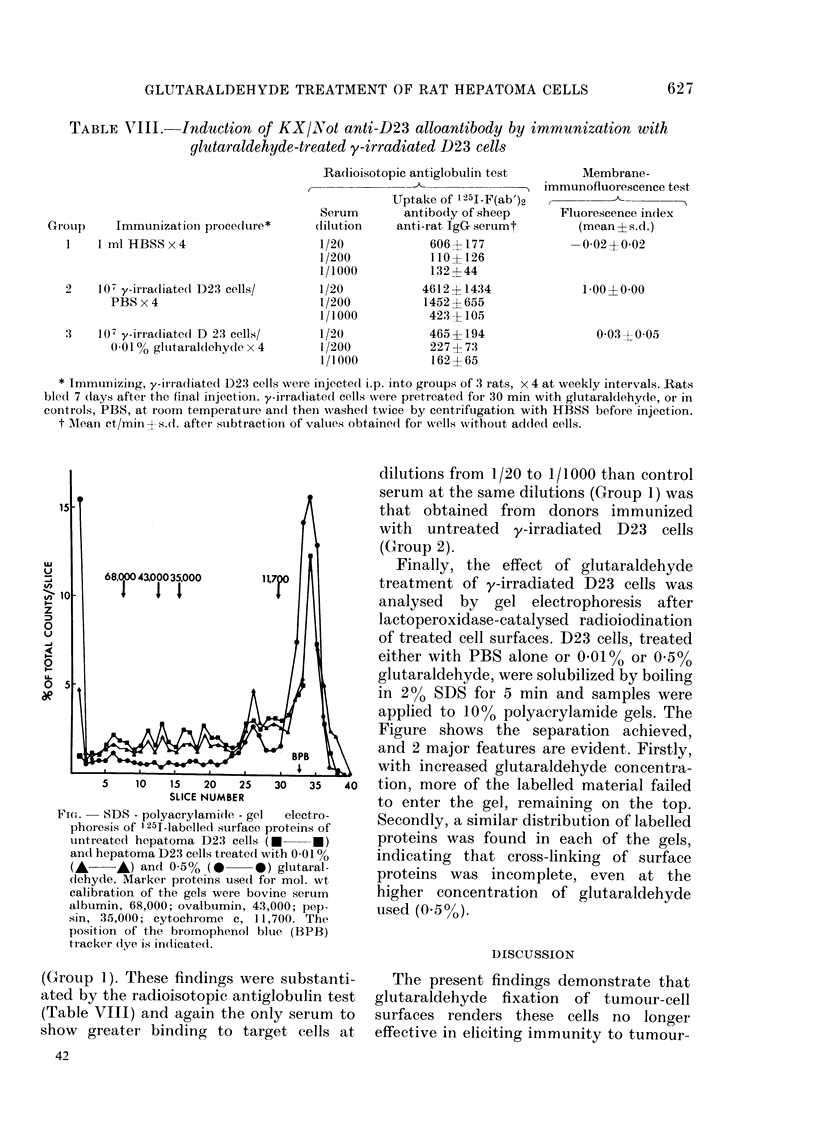

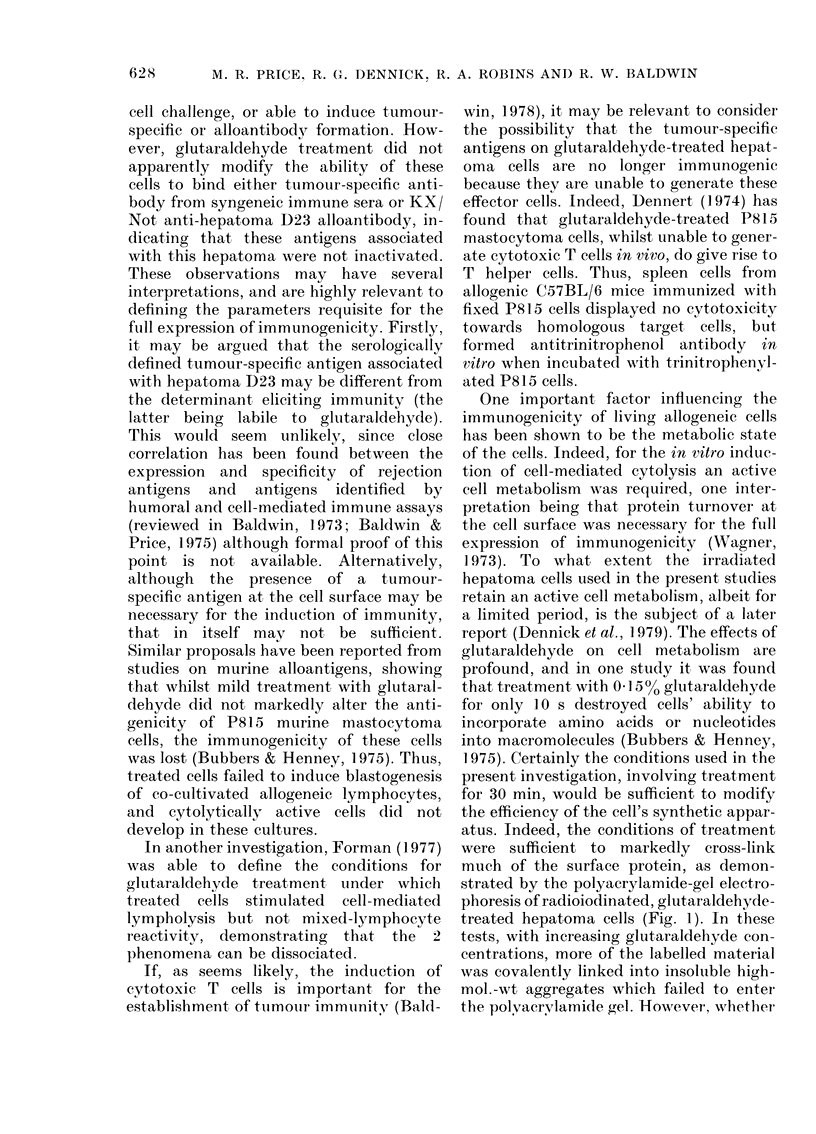

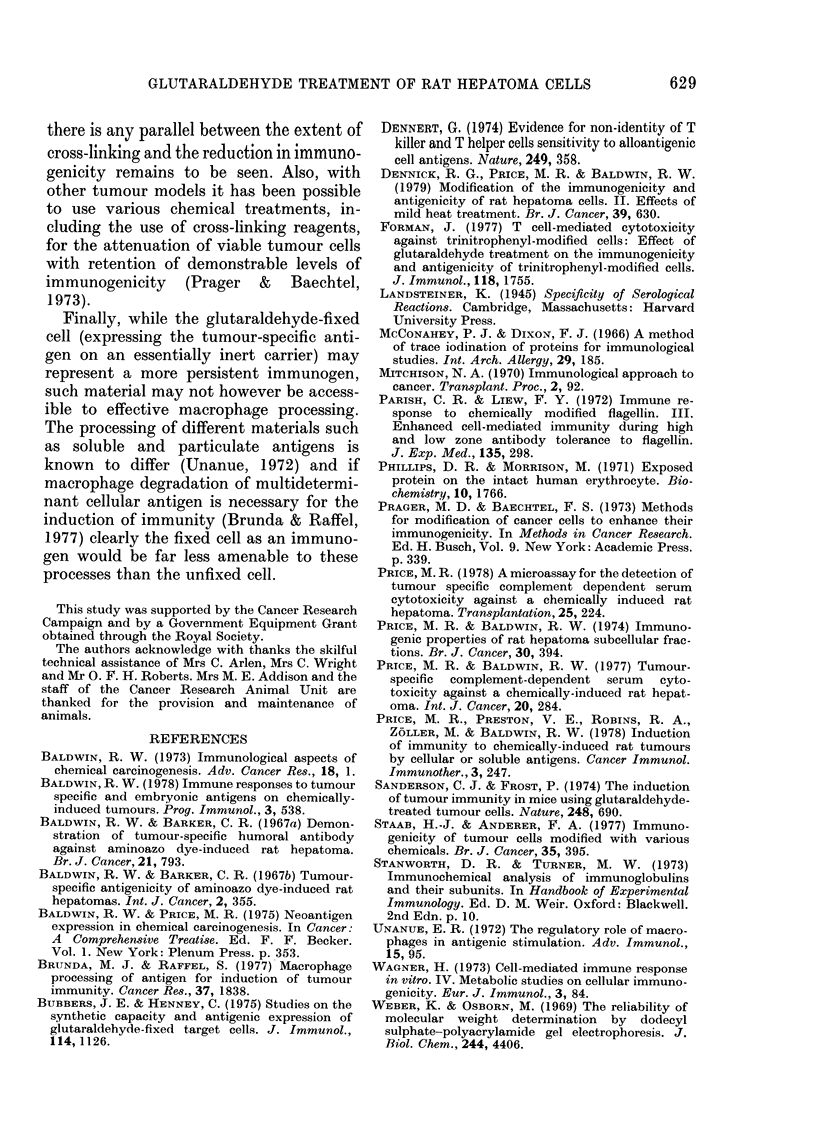


## References

[OCR_01118] Baldwin R. W., Barker C. R. (1967). Demonstration of tumour-specific humoral antibody against aminoazo dye-induced rat hepatomata.. Br J Cancer.

[OCR_01124] Baldwin R. W., Barker C. R. (1967). Tumour-specific antigenicity of aminoazo-dye-induced rat hepatomas.. Int J Cancer.

[OCR_01110] Baldwin R. W. (1973). Immunological aspects of chemical carcinogenesis.. Adv Cancer Res.

[OCR_01135] Brunda M. J., Raffel S. (1977). Macrophage processing of antigen for induction of tumor immunity.. Cancer Res.

[OCR_01140] Bubbers J. E., Henney C. S. (1975). Studies on the synthetic capacity and antigenic expression of glutaraldehyde-fixed target cells.. J Immunol.

[OCR_01146] Dennert G. (1974). Evidence for non-identity of T killer and T helper cells sensitised to allogeneic cell antigens.. Nature.

[OCR_01151] Dennick R. G., Price M. R., Baldwin R. W. (1979). Modification of the immunogenicity and antigenicity of rat hepatoma cells. II. Mild heat treatment.. Br J Cancer.

[OCR_01157] Forman J. (1977). T cell-mediated cytotoxicity against trinitrophenyl-modified cells: effect of glutaraldehyde treatment on the immunogenicity and antigenicity of trinitrophenyl-modified cells.. J Immunol.

[OCR_01169] McConahey P. J., Dixon F. J. (1966). A method of trace iodination of proteins for immunologic studies.. Int Arch Allergy Appl Immunol.

[OCR_01174] Mitchison N. A. (1970). Immunologic approach to cancer.. Transplant Proc.

[OCR_01178] Parish C. R., Liew F. Y. (1972). Immune response to chemically modified flagellin. 3. Enhanced cell-mediated immunity during high and low zone antibody tolerance to flagellin.. J Exp Med.

[OCR_01185] Phillips D. R., Morrison M. (1971). Exposed protein on the intact human erythrocyte.. Biochemistry.

[OCR_01197] Price M. R. (1978). A microassay for the detection of tumour-specific complement-dependent serum cytotoxicity against a chemically induced rat hepatoma.. Transplantation.

[OCR_01203] Price M. R., Baldwin R. W. (1974). Immunogenic properties of rat hepatoma subcellular fractions.. Br J Cancer.

[OCR_01208] Price M. R., Baldwin R. W. (1977). Tumour-specific complement-dependent serum cytotoxicity against a chemically induced rat hepatoma.. Int J Cancer.

[OCR_01221] Sanderson C. J., Frost P. (1974). The induction of tumour immunity in mice using glutaraldehyde-treated tumor cells.. Nature.

[OCR_01226] Staab H. J., Anderer F. A. (1977). Immunogenicity of tumour cells modified with various chemicals.. Br J Cancer.

[OCR_01238] Unanue E. R. (1972). The regulatory role of macrophages in antigenic stimulation.. Adv Immunol.

[OCR_01243] Wagner H. (1973). Cell-mediated immune response in vitro. IV. Metabolic studies on cellular immunogenicity.. Eur J Immunol.

[OCR_01248] Weber K., Osborn M. (1969). The reliability of molecular weight determinations by dodecyl sulfate-polyacrylamide gel electrophoresis.. J Biol Chem.

